# Effect of Different Proportions of Three Microbial Agents on Ammonia Mitigation during the Composting of Layer Manure

**DOI:** 10.3390/molecules24132513

**Published:** 2019-07-09

**Authors:** Shizheng Zhou, Xinyi Zhang, Xindi Liao, Yinbao Wu, Jiandui Mi, Yan Wang

**Affiliations:** 1 National Engineering Research Center for Breeding Swine Industry, College of Animal Science, South China Agricultural University, Wushan Road, Tianhe District, Guangzhou 510642, China; 2 Guangdong Provincial Key Lab of Agro-Animal Genomics and Molecular Breeding and Key lab of Chicken Genetics, Breeding and reproduction, Ministry of Agriculture, Guangzhou 510642, China

**Keywords:** ammonia migration, composting, layer manure, *Bacillus subtilis*, *Bacillus stearothermophilus*, *Candida utilis*

## Abstract

Odor emissions represent one of the important issues of aerobic composting. The addition of microbial agents to compost is an important method for solving this problem, but this process is often unstable when a single microbial agent is added to the compost. Therefore, in this study, five treatments comprising different proportions of *Bacillus stearothermophilus*, *Candida utilis*, and *Bacillus subtilis* were tested to determine the best combination of the three microbial agents for ammonia reduction, as follows: control group (CK), 2:1:1 (A), 1:1:2 (B), 1:2:1 (C), and 1:1:1 (D). Compared with the CK group, the A, B, C, and D groups reduced ammonia emissions by 17.02, 9.68, 53.11, and 46.23%, respectively. The total ammonia emissions were significantly lower in C and D than in CK (*p* < 0.05). These two treatment groups had significantly increased nitrate nitrogen concentrations and decreased pH values and ammonium nitrogen concentrations (*p* < 0.05). Throughout the composting process, the total bacterial number was significantly higher in C and D than in CK (*p* < 0.05). Therefore, it is likely that *B. stearothermophilus*, *C. utilis*, and *B. subtilis* compounded from 1:2:1 (C) to 1:1:1 (D) reduced the ammonia emissions due to (1) a reduction in the pH and (2) the promotion of the growth of ammonia-oxidizing bacteria and the conversion of ammonium nitrogen to nitrate nitrogen. This study provides a theoretical basis and technical support for the odor problem of layer manure compost and promotes the development of composting technology.

## 1. Introduction

In recent years, egg production and consumption have been increasing throughout the world. Since the 1980s, egg production in China has been the highest worldwide, accounting for approximately 40% of global production [1]. Laying hens are able to meet egg demand but simultaneously produce significant amounts of manure and waste.

To handle the excrement and waste, current intensive laying hen farms have primarily adopted high-temperature aerobic composting, anaerobic fermentation, feed treatment, and direct return to the field [2]. Of these treatments, high-temperature aerobic composting is a widely used, efficient, economical, and hygienic processing method [3].

Aerobic composting can not only solve the problem of waste pollution, but also transform the waste into fertilizer and make rational use of nutrients in feces such as organic matter, nitrogen, and phosphorus [4]. Microorganisms continuously catabolize organic compounds, producing many metabolites, with some of the intermediates and final products emitting volatile odors [5,6]. The inhalation of ammonia, amines, and mercaptans, and other odors can cause nausea, vomiting, dizziness, and other symptoms, which can lead to respiratory and nervous system poisoning [7]. According to statistics, malnutrition complaints about livestock and poultry farms in China are continuing to increase, ranking as the second most frequent type of farm-related complaint case behind sewage. Thus, malnutrition has become widely accepted as a valid concern [8]. Additionally, the volatilization of odors can lead to a loss of nutrients in the composting process, thereby reducing the value of the compost [9]. Therefore, a process to reduce laying hens’ odor emissions needs to be identified.

Of the various malodorous gases produced during layer manure composting, ammonia is one of the most important. First, during composting, ammonia is the most concentrated odor [2], with a previous study showing that its production can reach 17.347 mg/m^3^ when composting with chicken manure as the primary raw material [10]. Second, a significant positive correlation was observed between the amount of ammonia released and the concentration of other odoriferous compounds during composting [11]. Therefore, the ammonia concentration is often used to measure the level of odor emissions. Third, ammonia volatilization leads to the loss of nitrogen and affects compost nutrition [12]. Barrington et al. [13] showed that ammonia volatilization during composting is the primary cause of nitrogen loss in piles and accounts for more than 90% of total losses. Finally, in addition to odor, ammonia also catalyzes the formation of acid rain [14], which produces adverse effects, such as reducing the life of compost equipment [15]. Therefore, the reduction of ammonia emissions in the compost process is very important.

It has been reported that *Bacillus stearothermophilus*, *Bacillus subtilis*, and *Candida utilis* have the potential to reduce ammonia emissions during composting [16]. *B. stearothermophilus* is an aerobic or facultative anaerobic bacterium, with an optimum temperature of growth from 55–65 °C and its survival range is 30–75 °C [17]. *B. stearothermophilus* can ferment organic acids and small molecule carbohydrates such as glucose and lactose to reduce the pH and is expected to reduce ammonia emissions [18,19]. *B. subtilis* is an aerobic or facultative anaerobe with an optimum growth temperature of 37 °C. Since *B. subtilis* is an aerobic bacterium, it leads to bio-oxidative oxygen abatement. Thus, it can reduce the pH while consuming oxygen, creating the environment required for beneficial anaerobic bacteria, such as Bifidobacterium, to regulate the microecology [20]. *C. utilis* is an aerobic or facultative anaerobic fungus with an optimum growth temperature of 20–30 °C that decomposes sugars into carbon dioxide and water under aerobic conditions. In the absence of oxygen, the sugars are broken down into alcohol and carbon dioxide. The amount of crude protein in fermentation products increases with the addition of yeast (*p* < 0.05), indicating that *C. utilis* has a strong ability to use that nitrogen to synthesize fungal proteins, and the nitrogen-holding effect is obvious [21]. 

Because of the complexity of the microbial population in a heap, it is often difficult for a bacterium to become dominant following the addition of a single fungicide to the compost [22]. However, when the bacteria strains are compounded, the bacteria are more able to adapt to the compost environment compared with when a single bacterium is added. It is also easier for a bacterium to become the dominant strain, enabling it to display its effectiveness [23]. Therefore, bacteria compounds are commonly used to improve their functioning in the environment.

In this study, *B. stearothermophilus*, *B. subtilis*, and *C. utilis* were chosen as model microbes to identify the best mixture ratio for ammonia reduction during layer manure composting. The relevant physical and chemical indicators were analyzed to explore the compost process and the corresponding mechanisms of ammonia mitigation. This study might provide new ideas for the preservation of nitrogen and reduction of ammonia emissions in the process of layer manure composting.

## 2. Results

### 2.1. Ammonia Emissions

**Figure 1:**
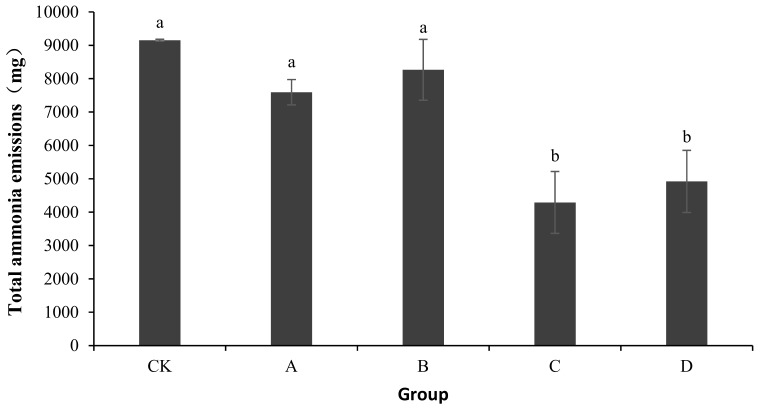
Change in total ammonia emissions during laboratory composting of the layer manure mixture. Note: The ratio of *Bacillus stearothermophilus*, *Candida utilis* and *Bacillus subtilis* in group A, B, C and D are 2:1:1, 1:1:2, 1:2:1, 1:1:1; ^a^ showed no significant differences in ammonia emissions among A, B, and CK (*p* > 0.05); ^b^ shows compared with CK, the addition of bactericides in C and D significantly reduced the ammonia release from the composting body (*p* < 0.05).

As shown in Figure 1, the total ammonia emissions of CK, A, B, C, and D were 9151.44 ± 29.51, 7593.53 ± 379.76, 8265.83 ± 913.32, 4291.19 ± 926.54, and 4920.68 ± 930.74 mg, respectively. Compared with CK, the ammonia emissions of A, B, C, and D reduced by 17.02, 9.68, 53.11, and 46.23%, respectively. An analysis of the variance showed no significant differences in ammonia emissions among A, B, and CK (*p* > 0.05). Compared with CK, the addition of bactericides in C and D significantly reduced the ammonia release from the composting body (*p* < 0.05).

**Figure 2:**
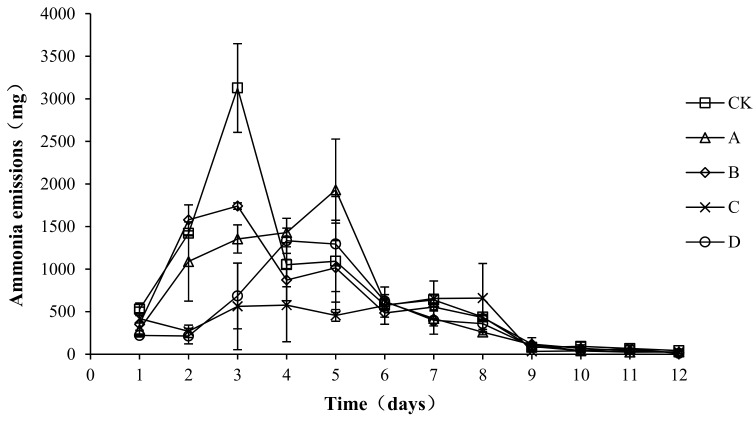
Periodic change in ammonia emissions during laboratory composting of the layer manure mixture. Note: The ratio of *Bacillus stearothermophilus*, *Candida utilis* and *Bacillus subtilis* in group A, B, C and D are 2:1:1, 1:1:2, 1:2:1, 1:1:1.

As shown in Figure 2, in all groups, the ammonia emissions showed a trend of first increasing, then decreasing, and then gradually becoming stable. The peak value of ammonia emissions of groups CK (3126.80 ± 520.68 mg/d) and B (1740.11 ± 37.77 mg/d) occurred on the third day, the peak value of ammonia emissions of group A (1930.76 ± 596.32 mg/d) occurred on the fifth day, and the peak value of ammonia emissions of groups C (576.44 ± 429.59 mg/d) and D (1332.73 ± 148.38 mg/d ) occurred on the fourth day. The ammonia emissions in the warming and high-temperature period (days 1–8) of samples CK, A, B, C, and D accounted for 96.93, 97.30, 96.57, 97.07, and 96.12% of the total ammonia emissions, respectively. An analysis of the variance showed that the peak values of ammonia emissions of A, B, C, and D were significantly lower than those of CK (*p* < 0.05), with the peak value of C being the lowest, significantly lower than that of group A (*p* < 0.05). No significant differences in ammonia emissions were detected among the CK, A, and B groups (*p* > 0.05) during the composting process, and ammonia emissions in the C and D groups were significantly lower than those in the other three groups (*p* < 0.05). Ammonia emissions were not significantly different between groups C and D (*p* > 0.05).

### 2.2. Variation in Temperature during Composting

**Figure 3:**
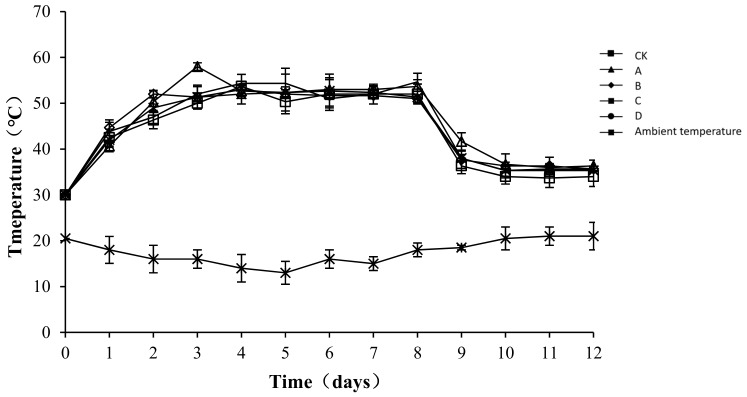
Periodic change in temperature during laboratory composting of the layer manure mixture. Note: The ratio of *Bacillus stearothermophilus*, *Candida utilis* and *Bacillus subtilis* in group A, B, C and D are 2:1:1, 1:1:2, 1:2:1, 1:1:1.

The temperature of compost is an indicator of microbial metabolic activity [24]. Moreover, microbial activity correlates positively with the content of organic matter that can be oxidatively decomposed in sediments, which indirectly reflects the efficiency of the composting process [25]. The temperature changes in each group and the ambient changes during the test are shown in Figure 3. The composting process involved a complete phase of warming up until a high temperature was reached, and then cooling occurred. Similar temperature changes occurred in the different treatment groups, with the peak level maintained for approximately one week, followed by a steady cooling down period. From the third day of composting, the temperature of the compost body was higher than 50 °C in all treatment groups. The highest temperature of the compost was 53.67 ± 2.62, 58.00 ± 0.82, 53 ± 0.82, 52.67 ± 3.68, and 54.67 ± 0.47 °C in CK, A, B, C, and D, respectively. After the 8th day, the temperature of all groups began to drop. An analysis of the variance showed that throughout the composting process, the temperature of the compost body of group CK was significantly lower than that of other groups (*p* < 0.05), but no significant difference was detected among the other groups (*p* > 0.05). As a possible reason for the difference, the microbial flora was relatively enriched after the addition of the bacteriocins. The growth, breeding, and metabolism produced biothermal energy, resulting in a slight increase in the temperature of the pile body.

### 2.3. Variation in pH during Composting

**Figure 4:**
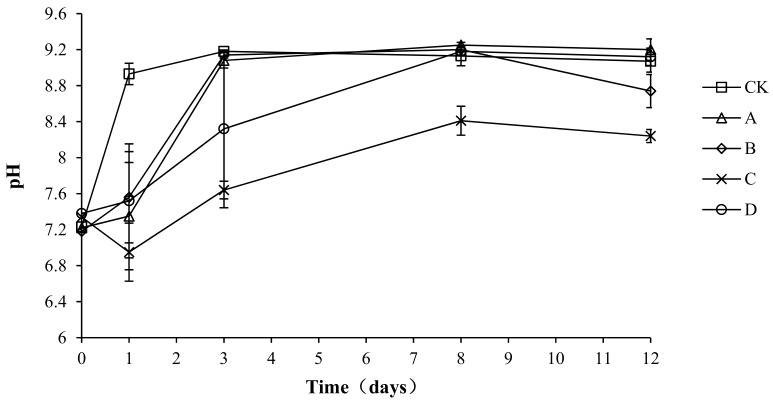
Periodic change in pH during laboratory composting of the layer manure mixture. Note: The ratio of *Bacillus stearothermophilus*, *Candida utilis* and *Bacillus subtilis* in group A, B, C and D are 2:1:1, 1:1:2, 1:2:1, 1:1:1.

The pH of the compost body not only has an important influence on the metabolic activity of the microorganisms, but also affects the activity of various enzymes and changes the rate of degradation reactions [26]. Neutral or weakly alkaline pHs are most suitable for the growth and reproduction of microorganisms, whereas these parameters are adversely affected by very high or very low pH levels. As shown in Figure 4, the pH of all groups was between 6.95 and 9.25, with a similar overall trend: a rapid increase followed by a stable state. The reason for this pattern could be related to the high nutrition content of the compost body during pre-composting, which allowed microbes to grow and reproduce rapidly, leading to the decomposition of nitrogen compounds and resulting in an increase in ammonium nitrogen. The ammonium nitrogen would react with water, resulting in an increase in OH^−^ ions and thus increasing the pH. Later, as the temperature decreased, nitrifying bacteria, whose growth was inhibited by high temperatures during the early stage, would multiply rapidly at a suitable temperature and undergo nitrification, which would result in an increased H^+^ concentration and explain the slight decrease or stabilization of pH values. The maximum pH values of CK, A, B, C, and D were 9.18 ± 0.01, 9.25 ± 0.03, 9.20 ± 0.02, 8.41 ± 0.16, and 9.18 ± 0.04, respectively. The minimum pH values of groups CK, A, B, C, and D were 7.64 ± 0.02, 7.35 ± 0.03, 7.56 ± 0.02, 6.95 ± 0.02, and 7.52 ± 0.03, respectively. The analysis of variance showed that the pH of group C was significantly lower than that of the other groups (*p* < 0.05) during the composting process, but no significant difference was found among the other groups (*p* > 0.05).

### 2.4. Variation in Moisture Content during Composting

**Figure 5:**
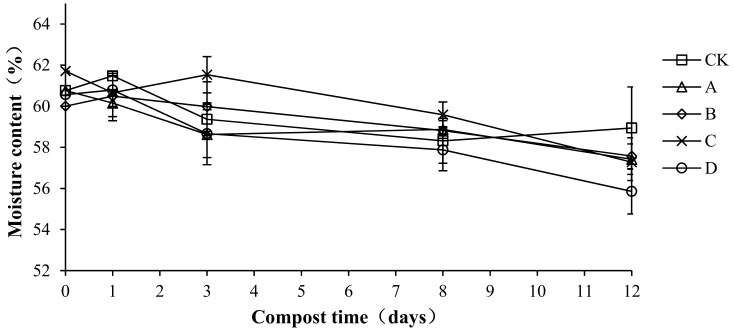
Periodic change in moisture content during laboratory composting of the layer manure mixture. Note: The ratio of *Bacillus stearothermophilus*, *Candida utilis* and *Bacillus subtilis* in group A, B, C and D are 2:1:1, 1:1:2, 1:2:1, 1:1:1.

Moisture is an indispensable condition for the growth and reproduction of microorganisms that is important for the decomposition of organic matter. The moisture content directly affects the speed of compost fermentation. A suitable water content provides a good environment for compost fermentation and is thus an important indicator of composting [27]. Moisture content changes with different proportions of compound bacteria are shown in Figure 5. The moisture content of each treatment group was maintained at approximately 60%, and the overall trend showed a slight decrease. In the compost body, water evaporates continuously and is lost as vapor, which could explain the decrease [28]. The initial water content of CK, A, B, C, and D was 60.76 ± 0.45, 60.76 ± 0.53, 60.01 ± 0.34, 61.71 ± 0.64, and 60.57 ± 0.47%, respectively. Samples of the average moisture content were 59.53 ± 0.94, 58.77 ± 0.55, 59.22 ± 0.12, 59.76 ± 0.10, and 58.30 ± 1.95%, respectively. There were no significant differences in the average moisture content among treatments (*p* > 0.05), which indicates that the addition of compound antibacterial agents had no effect on the moisture content of the compost body.

### 2.5. Variation in Organic Matter during Composting

**Figure 6:**
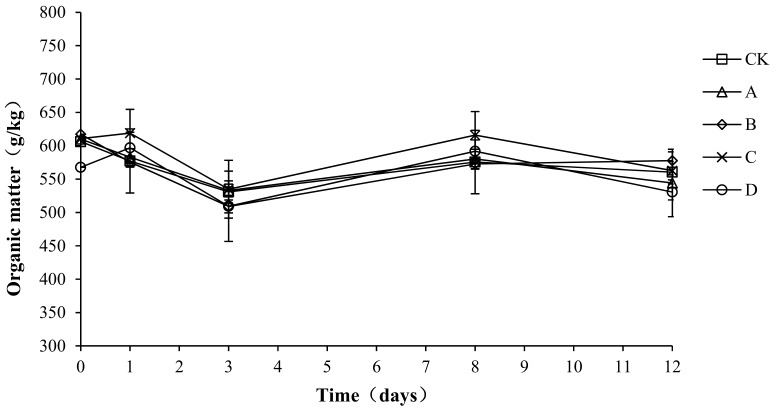
Periodic change in organic matter during laboratory composting of the layer manure mixture. Note: The ratio of *Bacillus stearothermophilus*, *Candida utilis* and *Bacillus subtilis* in group A, B, C and D are 2:1:1, 1:1:2, 1:2:1, 1:1:1.

Organic matter provides the carbon source for microbial aerobic fermentation. During the composting process, the organic matter continuously degrades, providing the microorganisms with energy, which enables them to grow and multiply rapidly. The organic matter forms humus after a complex degradation process, which increases the fertility of the compost body. As shown in Figure 6, the trend for organic matter in the compost process was basically the same for each group: a decrease first, then a rebound, and then a stable state. The initial organic matter content of CK, A, B, C, and D was 606.23 ± 5.23, 580.00 ± 3.48, 617.37 ± 5.68, 610.69 ± 4.33, and 567.69 ± 3.89 g/kg, respectively. At the end of composting, the organic matter of CK, A, B, C, and D decreased by 7.55, 6.17, 6.43, 7.86, and 6.52%, respectively.

An analysis of the variance showed no significant difference in organic matter loss among the different groups (*p* > 0.05). To possibly explain this result, the proportion of bacteria added to the compost body was 1.5%, which had little effect on the inherent flora of the compost body. Therefore, the compound bacteria did not play a role in promoting the degradation of organic matter.

### 2.6. Variation in Total Nitrogen during Composting

**Figure 7:**
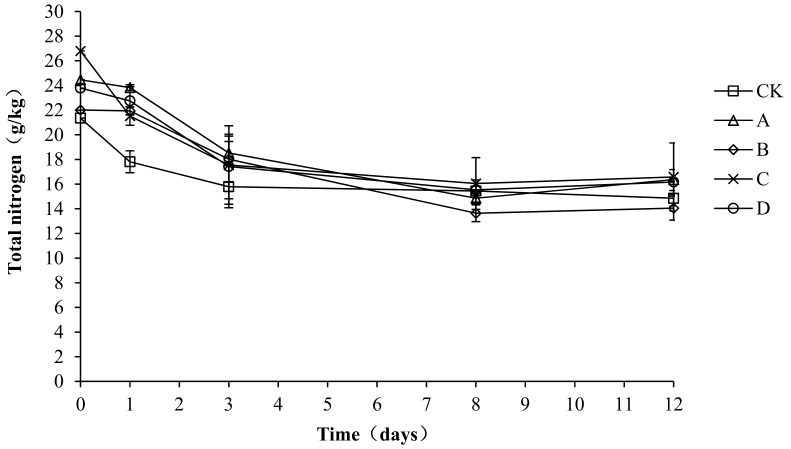
Periodic change in total nitrogen concentration during laboratory composting of the layer manure mixture. Note: The ratio of *Bacillus stearothermophilus*, *Candida utilis* and *Bacillus subtilis* in group A, B, C and D are 2:1:1, 1:1:2, 1:2:1, 1:1:1.

A nitrogen source is required for the microbial fermentation of nutrients. Both the synthesis of bacterial proteins and the provision of microbial energy require nitrogen sources. As shown in Figure 7, the trend of total nitrogen was the same in each group, with all showing a gradual decrease and then stabilization. The total nitrogen content of all treatment groups increased to the highest level in the early stage of composting, with the highest value in CK, A, B, C, and D being 23.67, 24.36, 22.65, 23.60, and 23.54 g/kg, respectively. At the end of composting, the total nitrogen content in CK, A, B, C, and D was 14.52, 16.37, 14.05, 18.37, and 17.50 g/kg, respectively. The total nitrogen loss rate of CK, A, B, C, and D was 38.66, 32.80, 37.97, 22.16, and 25.66%, respectively. Throughout the compost process, particularly during days 0–3, CK had a significantly lower total nitrogen content than A, C, and D (*p* < 0.05), which indicates that the agents had a certain nitrogen fixation effect during the warming up stage. No significant differences were detected among the groups from day 4 to the end of the experiment (*p* > 0.05).

### 2.7. Variation in Ammonium Nitrogen during Composting

**Figure 8:**
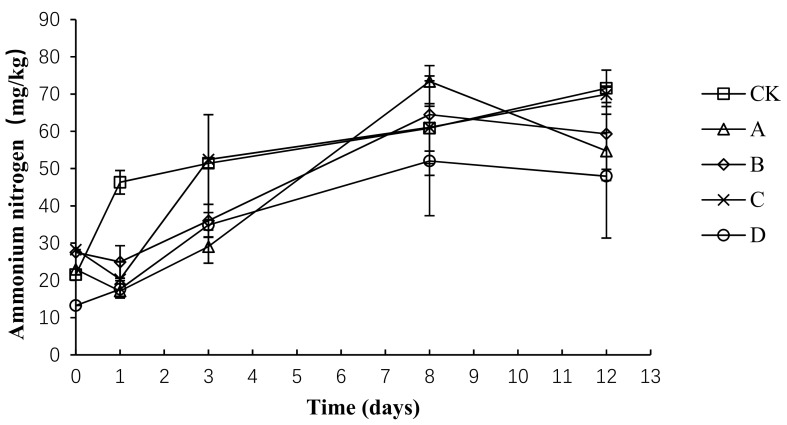
Periodic change in ammonium nitrogen concentration during laboratory composting of the layer manure mixture. Note: The ratio of *Bacillus stearothermophilus*, *Candida utilis* and *Bacillus subtilis* in group A, B, C and D are 2:1:1, 1:1:2, 1:2:1, 1:1:1.

The total nitrogen loss and ammonia emissions are primarily caused by the conversion of ammonium nitrogen into ammonia gas. As shown in Figure 8, the trend of ammonium nitrogen was the same in each group, showing an upward trend before the 8th day and then remaining relatively stable. The average content of ammonium nitrogen in CK, A, B, C, and D was 50.34 ± 16.77, 39.45 ± 21.27, 42.45 ± 16.38, 46.40 ± 19.04, and 33.14 ± 15.59 mg/kg, respectively. An analysis of the variance showed that the ammonium nitrogen concentration was significantly lower in group D than in group CK (*p* < 0.05) throughout the composting process, whereas no significant difference was detected among the other groups (*p* > 0.05).

### 2.8. Variation in Nitrate Nitrogen during Composting

**Figure 9:**
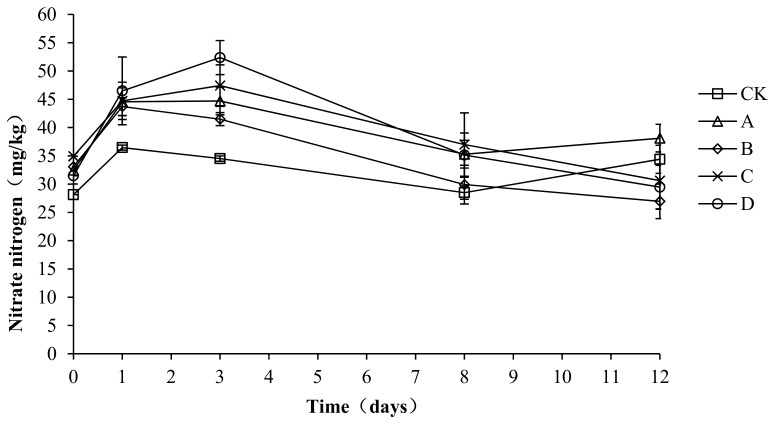
Periodic change in nitrate nitrogen concentration during laboratory composting of the layer manure mixture. Note: The ratio of *Bacillus stearothermophilus*, *Candida utilis* and *Bacillus subtilis* in group A, B, C and D are 2:1:1, 1:1:2, 1:2:1, 1:1:1.

As shown in Figure 9, the nitrate nitrogen first increased, then decreased, and finally remained steady. The average nitrate nitrogen content in CK, A, B, C, and D was 32.39 ± 3.41, 39.03 ± 4.91, 35.03 ± 6.52, 38.93 ± 6.24, and 38.98 ± 8.92 mg/kg, respectively. An analysis of the variance showed that the nitrate content was significantly lower in CK than in A, C, and D (*p* < 0.05) during the composting process, but no significant differences were detected among the other groups (*p* > 0.05). During the compost warming and high-temperature stage (days 0–8), the nitrate nitrogen content was significantly higher in C and D than in the other groups (*p* < 0.05). The above analysis shows that the compound fungicides in C and D could promote the compost process, particularly through nitrification in the warming and high-temperature stage, which increased the content of nitrate nitrogen.

### 2.9. Variation in the C/N Ratio during Composting

**Figure 10:**
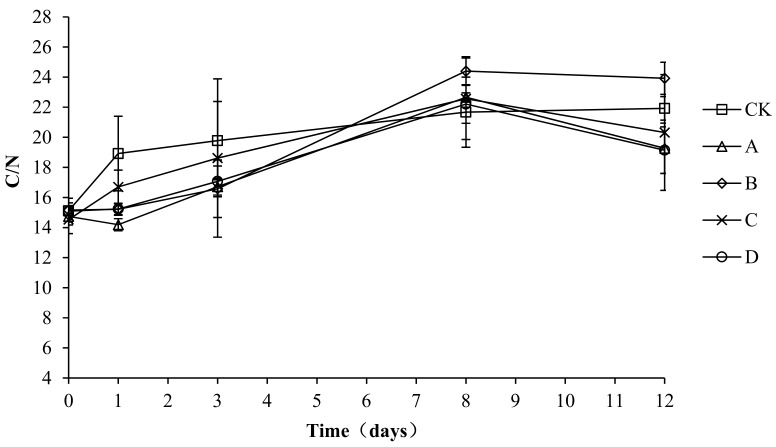
Periodic change in C/N concentration during laboratory composting of the layer manure mixture. Note: The ratio of *Bacillus stearothermophilus*, *Candida utilis* and *Bacillus subtilis* in group A, B, C and D are 2:1:1, 1:1:2, 1:2:1, 1:1:1.

Microbial metabolism for growth and reproduction requires carbon to provide energy, and both energy and nitrogen are required for the synthesis of cell protoplasts. When the C/N ratio of a compost body is too high, the relative excess carbon and lack of nitrogen lead to a retardation of microbial growth and a slowed composting process. When the C/N ratio of a compost body is too low, the nitrogen content is relatively higher than the carbon content, which may lead to the conversion of nitrogen to ammonia gas and volatilization, which causes nitrogen loss. The solid phase C/N ratio changes in each compost body are shown in Figure 10. The solid phase C/N ratio changes in each group are basically the same, with all increasing at first and then remaining stable. The initial C/N ratios of CK, A, B, C, and D were 15.11 ± 0.16, 14.74 ± 0.57, 15.16 ± 0.79, 14.53 ± 0.93, and 15.09 ± 0.55, respectively. At the end of composting, the C/N ratios of CK, A, B, C, and D were 21.92 ± 0.78, 19.27 ± 1.67, 23.92 ± 1.07, 20.32 ± 3.85, and 19.14 ± 1.55, respectively. An analysis of the variance showed no significant difference (*p* > 0.05) in the C/N ratio among the groups during the entire experiment, which indicated no significant effect of the composting agents on the C/N ratio and composting progress.

### 2.10. The Variation in Absolute Abundance of Total Bacteria (TB) in Different Groups

**Figure 11:**
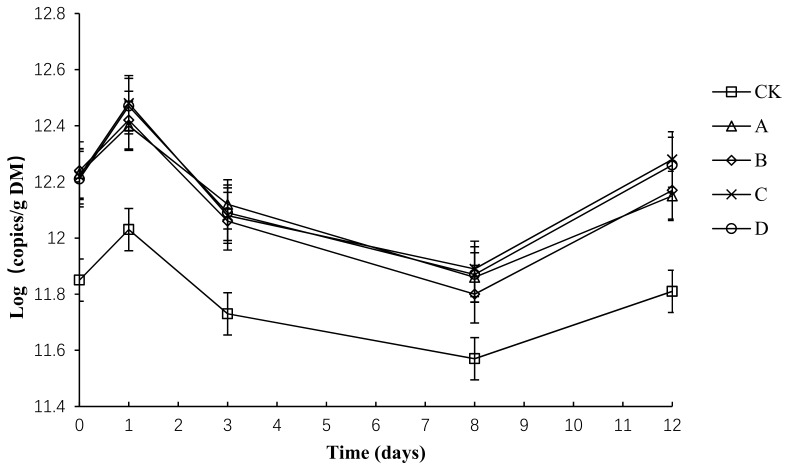
Total number of bacterial profiles for different composting treatments. Note: The ratio of *Bacillus stearothermophilus*, *Candida utilis* and *Bacillus subtilis* in group A, B, C and D are 2:1:1, 1:1:2, 1:2:1, 1:1:1.

The variation in the absolute abundance of TB in each group is shown in Figure 11. Throughout the compost process, the total number of bacteria in each group tended to be the same—a rapid rise and later rapid decline, before a final slight increase. Before the second day, the temperature rise was relatively slow, which is more suitable for bacterial growth and reproduction, so the total amount of bacteria increased slightly. By day 3, the composting had started to enter the high-temperature period. The growth of most heat-intolerant bacteria was inhibited, the thermophilic bacterial activity began to increase, and the total bacterial number decreased rapidly. After starting to cool down, the primary body of the thermophilic bacteria restored and reproduced. In this way, the total bacterial population increased.

Throughout the composting process, the TB abundance of each group was between 11.57 and 12.48. The initial number of total bacteria copies in CK, A, B, C and D had no significant difference (*p* > 0.05). By day 3, the number of total bacteria copies in CK, A, B, C and D peaked at the maximum. By day 12, the number of total bacteria copies in C and D had no significant difference but was significantly higher than that in CK, A and B (*p* < 0.05). During composting, the average numbers of total bacteria copies in CK, A, B, C and D was 11.80, 12.15, 12.14, 12.19 and 12.18, respectively, and the average number of total bacteria copies in A, B, C and D had no significant difference (*p* > 0.05), but was significantly higher than that in CK (*p* < 0.05).

### 2.11. The Variation in Absolute Abundance of Ammonia-Oxidizing Bacteria (AOB) in Different Groups

**Figure 12:**
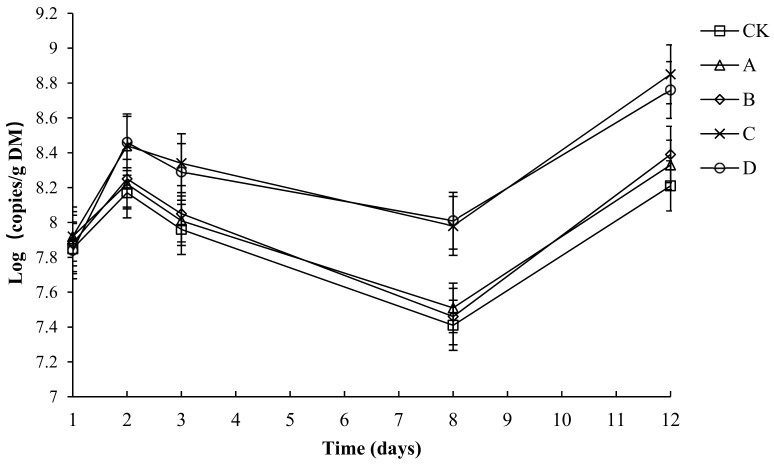
The number of ammonia-oxidizing bacteria (AOB) for different composting treatments. Note: The ratio of *Bacillus stearothermophilus*, *Candida utilis* and *Bacillus subtilis* in group A, B, C and D are 2:1:1, 1:1:2, 1:2:1, 1:1:1.

The variation in the absolute abundance of AOB in each group is shown in Figure 12. Throughout the compost process, the abundance of AOB in each group tended to be the same—a rapid rise and later rapid decline, before a final slight increase. Throughout the composting process, the AOB abundance of each group was between 7.41 and 8.84. The initial number of AOB copies in CK, A, B, C and D had no significant difference (*p* > 0.05). By day 3, the composting had started to enter the high-temperature period. The growth of AOB was inhibited. By day 8, after starting to cool down, the number of AOB copies in C and D had no significant difference but was significantly higher than that in CK, A and B (*p* < 0.05). By day 12, the number of AOB copies in C and D had no significant difference but was significantly higher than that in CK, A and B (*p* < 0.05). During composting, the average numbers of AOB copies in CK, A, B, C and D was 7.92, 8.00, 8.01, 8.31 and 8.27, respectively, and the average number of AOB copies in C and D had no significant difference (*p* > 0.05), but was significantly higher than that in CK, A and B (*p* < 0.05).

## 3. Discussion

Because of the complexity of the microbial population in a heap, it is often difficult for a bacterium to become dominant when adding a single fungicide to the compost [22]. In this study, the total ammonia emissions were significantly lower in C and D than in the CK group (*p* < 0.05). An analysis of physical and chemical indicators showed that these two treatment groups displayed significantly increased temperatures and nitrate nitrogen concentrations and decreased pHs. 

The release of ammonia is greatly affected by temperature and pH [29]. At 25 °C and under standard atmospheric pressure, ammonia is dissolved in water at a ratio of 700:1, but as the temperature increases, the solubility of ammonia decreases rapidly and it is released as a gas [30]. Both lactic acid bacteria and *B. stearothermophilus* produce acid during composting, thereby reducing the pH of the heap. Yan et al. [31] added a liquid compound microbial agent to layer manure compost and found that, in the early stage of composting, the pH of the treatment group with the microbial agent added decreased by 0.2–0.3 and was significantly lower than that in the control group (*p* < 0.01). Therefore, a low pH might be one of the reasons for low ammonia emissions in treatment groups C and D. 

The level of nitrogen also has a large effect on the release of ammonia. The nitrogen in a heap is primarily in the form of ammonium nitrogen, nitrate nitrogen, organic nitrogen, and ammonia, and the ammonium nitrogen is easily converted to ammonia gas and released under high pH and temperature conditions [32]. Studies have shown a significant positive correlation between ammonium nitrogen content and ammonia gas release during composting. Since the total amount of nitrogen in a heap is certain, when the content of ammonium nitrogen, which is easily converted into ammonia gas, is lowered, and the contents of the relatively more stable organic nitrogen and nitrate nitrogen increase, the release of ammonia gas can be effectively reduced. Mao et al. [33] selected two strains of nitrogen-transforming bacteria and added them to compost in 0.3% proportions and found that the ammonium nitrogen content of the treated group was reduced by more than 50% compared with that in the blank control group. This study showed that the composite bacteria groups could reduce the ammonium nitrogen content (*p* < 0.05) and increase the accumulation of nitrate nitrogen (*p* < 0.05), promoting the conversion of ammonium nitrogen to nitrate nitrogen. *C. utilis* can synthesize single-cell proteins with ammonium salt in a heap, such as nitrates and urea [34], among others, and the amount of nitrogen in a heap is relative. The stable forms are preserved, and ammonia volatilization is suppressed. This outcome is consistent with the results for the composite bacteria in this study, showing reduced ammonia emissions from the compost of laying hens.

The NH_3_ oxidizing bacterial including ammonia oxidizing bacteria (AOB) is adequate to oxidize ammonium to nitrite. Meanwhile, the generated nitrousoxide is subsequently oxidized to nitrate by nitrite-oxidizing bacteria [35]. Yao et al. [36] has reported that NH_4_^+^ oxidation is a complex process in nitrification, where many metabolic/catabolic reactions occur. Jiang et al. [37] showed that under inoculation with a nitrogen turnover bacterial agent, NH_3_ emissions were reduced, appropriately increasing nitrogen. In this study, the average number of AOB copies in C and D had no significant difference (p > 0.05), but was significantly higher than that in CK, A and B (*p* < 0.05). Therefore, the lower ammonia emissions in C and D might be due to promoting the growth of ammonia-oxidizing bacteria.

## 4. Materials and Methods 

### 4.1. Composting Materials

Fresh layer manure was collected from the experimental farm of South China Agricultural University. The sawdust was purchased from a wood processing factory in Zengcheng and dried in sunshine before testing. The primary components of the composting materials are shown in Table 1. *Bacillus stearothermophilus*, *Candida utilis*, and *Bacillus subtilis* were purchased from Guangdong Microbial Culture Collection Center located in China. The numbers of the strains were GIM1.371 = ATCC7953, GIM2.8 = AS2.281, and GIM1.131 = AS1.921. The purchased *B. stearothermophilus* was dissolved in 0.5 mL of nutrient broth, inoculated on a nutrient agar medium, and incubated at a constant temperature of 55 °C. *C. utilis* was dissolved in 0.5 mL of wort broth and inoculated on a wort plate of agar basal at a constant temperature of 25 °C. *B. subtilis* was dissolved in 0.5 mL of Luria-Bertani Culture (LB) broth and inoculated on a LB agar medium at a constant culture temperature of 37 °C. The three strains were sub-cultured twice in succession to stabilize them. After the production of lyophilized powder, a small part of 40% glycerol and bacteria were mixed 1:1 and placed at −80 °C in a refrigerator for sterilization. The content of the freeze-dried powder of *B. stearothermophilus*, *C. utilis*, and *B. subtilis* was approximately 10^8^ Colony-Forming Units (CFU) per gram of dry powder.

### 4.2. Experimental Design

Sawdust was used as a carbon source conditioner for the layer manure composting test. The wet weight ratio of the layer manure and sawdust was 4.90:1. The initial weight of the pile was approximately 600 g, and the control compost initial C/N ratio was 15:1, with a moisture content of 65%, based on the measured values. The experiment was divided into five treatment groups, a blank control group and four experimental groups, with each group comprising three replicates. The tests were conducted using a simulated composting method with a test cycle of 12 days [38]. *B. stearothermophilus*, *C. utilis*, and *B. subtilis* were compounded in different proportions and divided into the following five treatments: blank control group (CK), 2:1:1 (A), 1:1:2 (B), 1:2:1 (C), and 1:1:1 (D). The amount of compound bacteria used in each group was 5%. The layer manure, sawdust, and biochar were thoroughly mixed, and the moisture content of the composting mixture was adjusted to an initial moisture content of between 60 and 70% (*w*/*w*, wet weight) with water. No further adjustment was made to the moisture content throughout the composting period. A whirlpool pump and a gas-flow meter were used to aerate the mixtures. Fresh air was continuously pumped into a tube at the bottom of each closed vessel at the rate of 0.02 m^3^·h^−1^·kg^−1^ (wet basis) to supply oxygen throughout the composting period [39,40,41,42]. The core temperature of each composting pile was recorded three times daily at 09:00, 16:00, and 22:00 using temperature sensors.

### 4.3. Sample Collection

The composite samples and gas samples were recorded on days 0, 1, 2, 3, 4, 5, 6, 7, 8, 10, 12, and 15. Each vessel had an automatic turning device to homogenize the materials, and each pile was turned 3 min before sampling every day [43]. The composting process was stopped after 15 days, when it was observed that the temperatures of all piles had slowly decreased to an ambient temperature level. Samples of approximately 100 g were collected by mixing subsamples from the upper, central, and lower portions of the compost after using the turning device to achieve high representativeness [44]. Representative samples from each reactor were divided into two parts. One part was immediately stored at 4 °C until analysis, while the other part was air-dried, passed through a 0.25-mm sieve, and stored in a desiccator for further analysis [45]. Each closed vessel was also fitted with a suction pump with the outlet connected to a shunt device to divide the exhaust air into two portions: one portion was directed into a bubble adsorption tube containing a sulfuric acid solution to collect NH_3_, and the remaining portion was directed into a storage bag (2 m^3^) for 24 h [43]. Approximately 1 L of the air collected in the storage gas bag was transferred using a pump (Type: H-3020, Huada-Electron, Guangzhou, China) into an aluminum foil gas bag after being repeatedly reversed and thoroughly mixed for determination of the CH_4_ concentration [43,46].

### 4.4. Analytical Methods

#### 4.4.1. Gaseous Measurements

The NH_3_ in the sulfuric acid solution was determined by the absorption of the sodium naphthalene by sulfuric acid.

#### 4.4.2. Physicochemical Analyses

The moisture content was determined by drying at 105 °C to a constant weight. The pH was measured in a 1:10 (*w*/*v*, dry weight basis) water-soluble extract using a pH meter [47]. NH_4_^+^ and NO_3_^−^ were extracted with 2 M KCl (1:10 ratio for 1 h) and subjected to colorimetric analysis [48]. The total nitrogen content was measured by the modified micro-Kjeldahl digestion method with an automatic Kjeldahl apparatus (KDY-9830, China) [49]. The organic matter (OM) and ash contents were measured with a muffle furnace at 550 °C for 6 h. The total carbon (TC) content (%) was calculated from the ash fraction (%ash) with the following equation: %C = (100 − %ash)100/1.8 [50]. The loss of OM was calculated from the initial (OM_i_) and final (OM_F_) OM contents, according to the following equation [51]: OM loss (%) = 100[100 (OM_i_ − OM_f_)]/[OM_i_ (100 − OM_f_)]. The C/N ratio was calculated as TC (%)/TN (%). The pile mass was based on the total mass of all composting materials, including layer manure, sawdust, and biochar, at the initial and final composting stages. Based on the N concentration and the total pile mass before and after composting, the actual N amount was calculated by multiplying the N concentration and the total pile mass of the composting material. The actual N loss was the difference between the initial and final N concentrations in the composting materials [52].

#### 4.4.3. Total DNA Extraction and qPCR

The microbial total DNA of the composting samples was extracted using an E. Z. N. A. TM Soil DNA Kit (Omega, Norcross, GA, USA). The compost sample total microbial DNA was subjected to an ordinary Polymerase Chain Reaction (PCR) reaction using specific primers. Quality of the DNA was checked through electrophoresis in 2% agarose gel. The qPCR was performed in triplicate on a real-time PCR instrument (7500, Applied Biosystems, Foster City, CA, USA) using primer sets AOB-F (5′-GGGGTTTCTACTGGTGGT-3′)/AOB-R (5′- CCCCTCKGSAAAGCCTTCTTC-3′) for ammonia-oxidizing bacteria (AOB) genes [53] and TB-F (5′-ATTACCGCGGCTGCTGG-3′)/TB-R (5′-CCTACGGGAGGCAGCAG-3′) for total bacteria (TB) genes [38]. Each 20 µL of qPCR reaction contained 10 µL of 2 × SYBR Green PCR Master mix (Qiagen, Shanghai, China), 0.5 µL of each AOB or Bacteria primer (10 µM), and 1 µL of DNA template (20 ng/ µL) and was adjusted with sterile water to volume. The amplification was conducted using an initial denaturation step at 95 °C for 10 min, followed by 40 cycles of 30 s at 95 °C, 30 s at 55 °C, and 30 s at 72 °C for AOB and an initial denaturation step at 94°C for 4 min, followed by 35 cycles of 30 s at 94 °C, 30 s at 63 °C, and 72 s at 72 °C for bacteria. Melting curve analysis was carried out to verify amplicon specificity. A negative control without DNA template was used in all of the qPCR amplifications. Standard curves were generated on the basis of serial 10-fold dilutions of plasmids containing cloned AOB and genes and ranged from 1.0 × 10^3^ to 1.0 × 10^8^ copies per assay. Amplification efficiencies of 97.88% (AOB) and 103.5% (TB) were obtained using the slopes -3.41 and -3.21 of the standard curve, respectively. Linearity (r^2^) for AOB and TB was 0.999 and 0.998, respectively. The TB and AOB were expressed as the logarithm of copies/g of dry matter (DM), more information can be found in the Appendix A.

## 5. Conclusions

In this study, *B. stearothermophilus*, *C. utilis*, and *B. subtilis* were added to manure compost in ratios of 2:1:1 (A), 1:1:2 (B), 1:2:1 (C), and 1:1:1 (D). The total ammonia emissions were significantly lower in groups C and D than in CK (*p* < 0.05). An analysis of physical and chemical indicators showed that the pH levels in C and D were significantly lower than those in CK (*p* <0.05), which might be one reason for the lower ammonia emissions in treatment groups C and D. The results also showed that the composite bacteria groups (C and D) were able to reduce the ammonium nitrogen content (*p* < 0.05) and increase the accumulation of nitrate nitrogen (*p* < 0.05). Another reason for the lower ammonia emissions in treatment groups C and D might have been the promotion of the conversion of ammonium nitrogen to nitrate nitrogen. Another reason for the lower ammonia emissions in C and D might be that it was due to promoting the growth of ammonia-oxidizing bacteria. This study provides a new solution to the odor problem caused by the composting of layer manure, thereby reducing the environmental impact of the development of animal husbandry.

## Figures and Tables

**Table 1:** The main ingredients of composting materials.

Group	Moisture Content (%)	Total Carbon (%)	Total Nitrogen (%)	C/N
Chicken Dung	66.76	35.27	4.33	8.15
Sawdust	8.98	57.82	0.30	192.73

**Figure A1:**
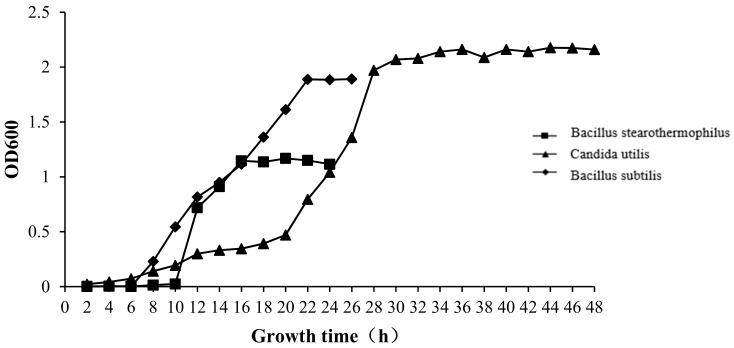
The growth curve of bacteria.

**Figure A2:**
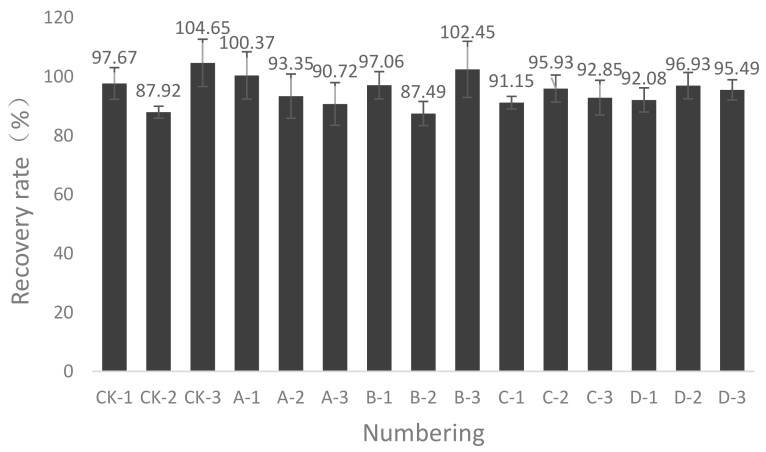
The 15 sets of the simulated composting equipment recovery rate.

## Appendix A

### Appendix A.1. Growth Curve Test of Strains

For the growth curves, 10 g of aseptic lyophilized powder of *B. stearothermophilus*, *C. utilis*, or *B. subtilis* was placed in 90 mL of sterilized saline (0.9%) in a sterilized flask, which was fully mixed evenly for a uniform 1:10 dilution. Using a 1 mL sterile pipette, 1 mL of diluent was slowly injected along the wall of a triangular flask containing 9 mL of sterile saline, which were mixed well to make a 1:100 dilution. Using another 1 mL sterile pipette, according to the above sequence of operations, 10-fold increasing dilutions were prepared (10^−2^ to 10^−7^). From each dilution, 100 μL was removed and inoculated on nutrient broth agar medium, wort agar medium, or LB agar medium plates (three replicates for each serial number), producing numbers at different dilutions. Plates were reversed. *B. stearothermophilus* was placed in a 55 °C incubator, *C. utilis* in a 25 °C incubator, and *B. subtilis* in a 37 °C incubator for 72 h to obtain pure culture samples. Single colonies of one pure culture sample were picked and inoculated into 50 mL of nutrient liquid medium, wort liquid medium, or LB liquid medium. *B. stearothermophilus* was cultured with shaking at 175 rpm for 48 h at 55 °C, *C. utilis* at 25 °C and 175 rpm for 48 h, and *B. subtilis* at 37 °C and 175 rpm for 48 h. Every 2 h, 2 mL of the culture media was placed in a cuvette, with a blank control of culture medium without a strain, and the optical density (OD) was tested at the wavelength of 600 nm by ultra-micro UV spectrophotometer (NanoDrop 2000c, Thermo, Waltham, MA, USA). The OD value represents the number of microorganisms [54].

The bacterial growth curves of the three strains are shown in Figure A1. *B. stearothermophilus* was in a stagnant growth phase in the first 10 h, entered a logarithmic growth phase from 10–16 h, and the growth then tended to stabilize. *C. utilis* grew slowly from 0–20 h, entered the logarithmic growth phase from 20–28 h, and the growth then tended to stabilize. *B. subtilis* was in a stagnant growth phase in the first 6 h, entered the logarithmic growth phase from 6–22 h, and the growth then tended to stabilize.

### Appendix A.2. Recovery Test

First, the tightness of the box device was checked, which was then connected to the pump, flow meter, sulfuric acid absorption solution, and other components. The pump was opened, and the pump suction rate was set to ensure that 15 sets of composting equipment exhaust rates remained at approximately 0.16 m^3^/h. The gas concentration was measured in the collection box and in the air. An ammonia tank was connected to a good valve, flow meter, and exhaust pipe. The first balance with the ammonia tank was weighing the initial weight and recording data. The simulation compost box was closed, the air pump was turned on, parameters were set, and air extraction began when the ammonia tank was opened with the flow meter activated and then the timing began. After a period of time, the entire tank system was close to equilibrium. For gas recovery, first, the pump was turned off and the ammonia valve was closed and, then, the weight of the ammonia tank was quickly weighed. Based on the amount of ammonia absorbed in the sulfuric acid absorption liquid, the recovery rate of the tank was calculated.

Figure A2 shows the measured results of 15 sets of simulated composting equipment recovery rates. For the 15 sets of simulated compost boxes used in this study, the recovery was measured between 87.49 and 104.65%. The average recovery was 95.07%, so all the equipment was considered airtight and reliable and could be used for the determination of ammonia emissions during composting.
